# Probiotic *Bifidobacterium* longum BB68S Improves Cognitive Functions in Healthy Older Adults: A Randomized, Double-Blind, Placebo-Controlled Trial

**DOI:** 10.3390/nu15010051

**Published:** 2022-12-22

**Authors:** Shaoqi Shi, Qi Zhang, Yue Sang, Shaoyang Ge, Qi Wang, Ran Wang, Jingjing He

**Affiliations:** 1 Key Laboratory of Functional Dairy, Co-constructed by Ministry of Education and Beijing Government, Department of Nutrition and Health, China Agricultural University, Beijing 100190, China; 2 Hebei Engineering Research Center of Animal Product, Langfang 065200, China; 3 Xinjiang Golden Camel Investment Co., Ltd., Wulumuqi 830039, China

**Keywords:** probiotic, cognitive function, healthy older adults, gut microbiota

## Abstract

Probiotics could improve cognitive functions in patients with neurological disorders such as Alzheimer’s disease, but the effects on cognitive function in healthy older adults without cognitive impairment need further study. The purpose of this study was to investigate the effect of *Bifidobacterium longum* BB68S (BB68S) on cognitive functions among healthy older adults without cognitive impairment. A randomized, double-blind, placebo-controlled trial was conducted with 60 healthy older adults without cognitive impairment who were divided into probiotic or placebo groups and required to consume either a sachet of probiotic (BB68S, 5 × 10^10^ CFU/sachet) or placebo once daily for 8 weeks. The Montreal Cognitive Assessment (MoCA) was used as an inclusion screening tool to screen elderly participants with healthy cognitive function in our study, and the Repeatable Battery for the Assessment of Neuropsychological Status (RBANS) was used to assess cognitive function in subjects before and after intervention as an assessment tool. BB68S significantly improved subjects’ cognitive functions (total RBANS score increased by 18.89 points after intervention, *p* < 0.0001), especially immediate memory, visuospatial/constructional, attention, and delayed memory domains. BB68S intervention increased the relative abundances of beneficial bacteria *Lachnospira*, *Bifidobacterium*, *Dorea*, and *Cellulosilyticum*, while decreasing those of bacteria related to cognition impairment, such as *Collinsella*, *Parabacteroides*, *Tyzzerella*, *Bilophila, unclassified_c_Negativicutes*, *Epulopiscium*, *Porphyromonas*, and *Granulicatella*. In conclusion, BB68S could improve cognitive functions in healthy elderly adults without cognitive impairment, along with having beneficial regulatory effects on their gut microbiota. This study supports probiotics as a strategy to promote healthy aging and advances cognitive aging research.

## 1. Introduction

Research in the field of aging and neuroscience reveals that the human brain shrinks with the process of normal aging, while the ventricles expand and both gray and white matter are reduced [1], resulting in alterations in neurological function that cause age-related cognitive decline [2,3]. As the population ages, a great deal of the elderly are at risk of age-related cognitive decline, which not only threatens their personal lives but also poses a challenge to society [4]. Therefore, it is necessary to develop efficacious interventions and preventative strategies for age-related cognitive decline and promote healthy aging of the global population.

The gut microbiota was first defined by Lederberg and McCray, accentuating the importance of microorganisms located in the human body in health and diseases [5]. Compelling evidence shows that the gut microbiota plays a critical role in powerfully modulating brain activity through the gut-brain axis [6,7,8]. Furthermore, owing to its reciprocal relationship with age, the gut microbiota changes during host aging, is altered in age-related diseases, and plays a role in modifying age-related health impairment of the host [9,10,11]. Accumulating evidence links the gut microbiota to the cognitive function of older adults. Regulating the gut microbiota as a prevention therapy for age-related cognitive decline has become an active research strategy.

Probiotics, live microorganisms that provide health benefits when administered regularly in the host, substantially affect the composition as well as metabolic output of the gut microbiome [12,13,14]. Probiotics can impact brain health and host behaviors through the microbiota-gut-brain axis [15,16,17]. Their use in the treatment of neurological diseases is being actively investigated [18,19,20]. Numerous animal studies have confirmed that probiotics can improve brain health, along with many human studies that have also explored the impact of probiotic intervention on cognitive function, which have achieved some positive results [21,22,23]. However, existing research has mainly focused on patients with cognitive dysfunction, such as mild cognitive impairment, Alzheimer’s disease, and severe depressive disorder [24,25]. Studies aimed at healthy elderly people without cognitive impairment are scarce. Therefore, more population studies targeting the healthy elderly are urgently needed to supplement evidence on the role of probiotics in improving the cognitive function of this specific population.

*Bifidobacterium longum* BB68S (BB68S, CGMCC No. 14168) is a potential probiotic. The 16S ribonucleic acid (rRNA) sequence of *B. longum* BB68S is available in the NCBI database under sequence number OP984807. Our previous in vivo and in vitro study evaluated and verified its safety [26]. In an intervention trial, we observed that BB68S can relieve constipation and regulate the gut microbiota [27]. Considering its other potential functions, we aimed to investigate the effects of BB68S in improving cognitive function in healthy older adults. We also focused on providing clinical evidence for validating the properties and effects of probiotics on cognitive aging and healthy aging, thereby supporting their better implementation in the expanding fields of healthcare and alternative medicine.

## 2. Methods

### 2.1. Study Design

**Figure 1:**
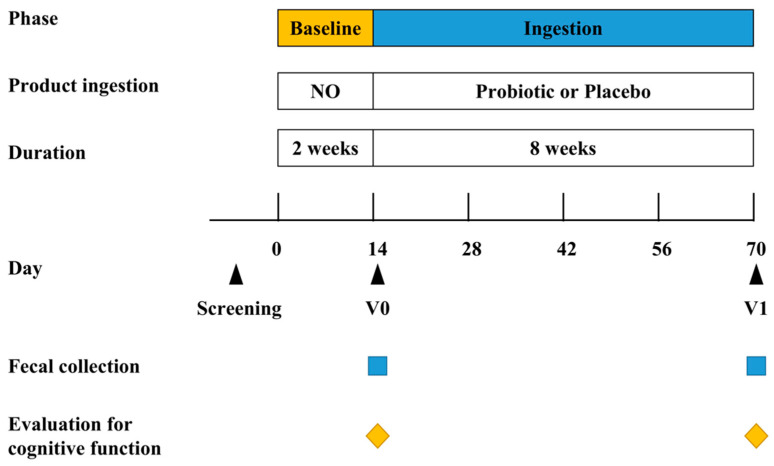
Study design. Probiotic: lyophilized powder containing *B. longum* BB68S. Placebo: maltodextrin. V0–V1: visit 0–visit 1.

To investigate the effects of BB68S on the cognitive function of healthy elderly people aged 60–75 years, we designed a randomized, double-blind, placebo-controlled trial. The sample size was estimated according to Assessment of Neurological Status (ANS) scores demonstrated by Xiao et al. [28]. We calculated that 30 subjects per group were needed with an α-error of 0.05 and β-error of 0.20. This 10-week study included a 2-week baseline period and an 8-week ingestion period (Figure 1). To remove the impact of any previous intake, a 2-week baseline period was required. The participants were solicited to not change their normal dietary patterns throughout the baseline period and were not allowed to consume probiotics or any dietary supplements with probiotics. On day 14 ± 1, their fecal samples were collected and the cognitive functions of the participants were evaluated. The participants were randomly divided into either the placebo or probiotic groups. During the ingestion period, the subjects were required to consume a sachet of probiotic (BB68S, 5 × 10^10^ CFU/sachet) or placebo (maltodextrin powder without probiotics) after lunch or dinner daily for 8 weeks. Throughout the intervention period, the subjects were asked to abstain from consuming any other probiotics or any dietary supplements with probiotics while they were required to be consistent with their daily diet and exercise, thus excluding their corresponding influence on the test results. To monitor the stability of their lifestyle, subjects were requested to record their daily dietary intake. The subjects received adequate training before recording in their daily diaries. In addition, any adverse events or symptoms of discomfort were also required to be recorded. On day 70 ± 1, upon the completion of the ingestion period, fecal samples were collected in a sterile tube and stored at −80 °C until further analyses. The cognitive functions of the subjects were again evaluated using the RBANS scores. The study was authorized by the China Agricultural University Ethics Committee (CAUHR-2021021) and filed under registration number ChiCTR2200062331 (http://www.chictr.org.cn, accessed on 21 November 2022). Written informed permission was acquired from each subject.

### 2.2. Participants

The subjects’ personal information was collected, including age, sex, disease status, and drug use in the first round of screening. Experienced researchers assessed their physical conditions based on medication history and dietary habits, and patients were screened according to the inclusion and exclusion criteria.

### 2.3. Inclusion and Exclusion Criteria

We selected subjects based on the following inclusion criteria: (i) aged 60–75 years at the time of screening and (ii) with healthy cognitive function judged by the Montreal Cognitive Assessment (MoCA) screening tool. The specific criteria were as follows: total MoCA scores were 19 or higher for those with 6 or fewer years of education, 22 or higher for those with 7–12 years of education, and 24 or higher for those with 12 or more years of education. In our study, we used the Chinese Beijing Version of MoCA (http://www.mocatest.org, accessed on 21 November 2022) to define the cognitive health of the elderly. The exclusion criteria were as follows: (i) obvious cognitive decline, or diagnosis with Alzheimer’s disease, mild cognitive impairment, etc.; (ii) hearing, visual, or communication disabilities or difficulties, incapable of living independently, or surgical history of digestive system resection; (iii) history or clinical trace of nervous system disease or psychosis; (iv) history of serious diseases, such as those of the heart, liver, kidney, or hematopoietic system; (v) use of the test-related product (other probiotic products or antibiotics, anti-inflammatory medications, gastrointestinal medicine) within a short period; (vi) unable to eat the test product according to the regulations, and (vii) participation in other clinical studies.

### 2.4. Randomization and Blinding

During the screening process, demographic data were collected, and the examination for cognitive ability assessment was conducted. The research assistant who was otherwise not involved in the study completed randomization using SAS program version 9.4 with Pocock and Simon minimum randomization methods and was required to conceal the randomization results from all researchers and subjects. The randomization method performed dynamic randomization to maximally balance the baseline characteristics between the two groups. The randomized data were stratified by factors including sex (male, female), age (60~64 years old, 65~69 years old, ≥75 years old), and MoCA scores (19~21, 22~24, ≥25). The subjects were evenly and randomly allocated to the probiotic or placebo groups. The research team members remained blind to each participant’s assigned group until all data were collected.

### 2.5. Fecal Sample Collection

Fecal samples were collected before and after intervention. We provided a cryogenic storage box (4 °C) and a sterile tube with a scoop inside the lid, instructing subjects to gather fecal samples into the tubes within 24 h before visiting and transporting them in the cryogenic storage box to the research site. The tubes were kept tightly sealed and stored in the cryogenic storage box until they were sent to the laboratory, following which, the samples were immediately stored at −80 °C until further analyses.

### 2.6. Evaluation of Cognitive Functions

Before and after intervention, we used the Repeatable Battery for the Assessment of Neuropsychological Status (RBANS) as an assessment tool to assess the subjects’ cognitive function. During the assessment, the subjects were placed in a quiet and undisturbed environment and simultaneously evaluated by two researchers experienced in using the evaluation form. One of the two researchers was required to speak uniformly and clearly to inform subjects of the description of each item, to ensure that they heard all instructions, and finally compute the average of the original scores of the 12 items recorded simultaneously by the two researchers. The twelve averages were then converted to five global domain scores as the final score for the corresponding subjects.

### 2.7. Gut Microbiota Analysis

#### 2.7.1. Genomic DNA Extraction, Amplification, and Sequencing

The bacterial genomic DNA was extracted from fecal samples according to the method described by Tang et al. [29]. Purity was determined by 1% agarose gel electrophoresis and high-quality DNA (OD260/280 ≥ 1.5, ≥ 150 ng) was amplified using the 338F-806R primer [30] in the hypervariable V3–V4 region of the bacterial 16S rRNA gene. High-quality PCR products were sequenced on the Illumina MiSeq PE300 platform.

#### 2.7.2. Processing of Sequencing Data

The original reads were demultiplexed and quality-filtered using the fastp 0.20.0 tool [31] and integrated using FLASH 1.2.7 [32]. UPARSE 7.1 (available at http://drive5.com/uparse/, accessed on 21 November 2022) was used to cluster the operational taxonomic units (OTUs) using a 97% similarity criterion [33], and chimeric sequences were discovered and eliminated. RDP Classifier version 2.2 [34] was used with a confidence threshold of 0.7 to evaluate the taxonomy of each OTU representative sequence according to the 16S rRNA database (Silva v138).

### 2.8. Statistical Analysis

When describing the baseline characteristics, we used the intent-to-treat dataset, comprising all subjects who underwent random assignment to a treatment group, and when analyzing the main results, the per-protocol set was adopted. Normality tests were performed on all continuous variables using the Kolmogorov-Smirnov test. We calculated means (SDs) for continuous variables and counts (percentages) for categorical variables. The independent samples *t*-test was used for the comparison of continuous variables and the chi-square test was used for the comparison of categorical variables. For the primary outcome, RBANS scores at week 8, an analysis of covariance model adjusted for baseline scores was used to examine the difference between the probiotic and placebo groups. With a significant threshold setting at *p* < 0.05, data analysis was performed using IBM SPSS statistics 23.0.

## 3. Results

### 3.1. Baseline Characteristics

**Figure 2:**
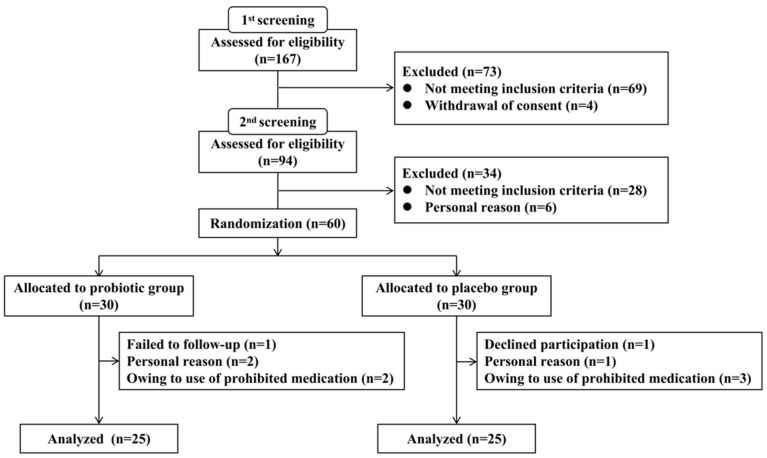
Trial profile.

A total of 167 subjects were recruited for screening, and 60 subjects were chosen based on the inclusion criteria. The subjects were randomized to give 30 subjects in each of the placebo and probiotic groups. Ten subjects withdrew their consent and dropped out, including five from each of the placebo and probiotic groups, with 50 subjects finally finishing the trial (Figure 2). The intervention was carried out with 83% compliance and no clinically significant adverse events occurred. Table 1 summarizes the demographic and clinical variables at baseline, including age, weight, BMI, sex, education level, MoCA score, and RBANS score. These characteristics were comparable between the groups (all *p*-values were greater than 0.05).

### 3.2. Primary Outcomes for Cognitive Function

**Figure 3:**
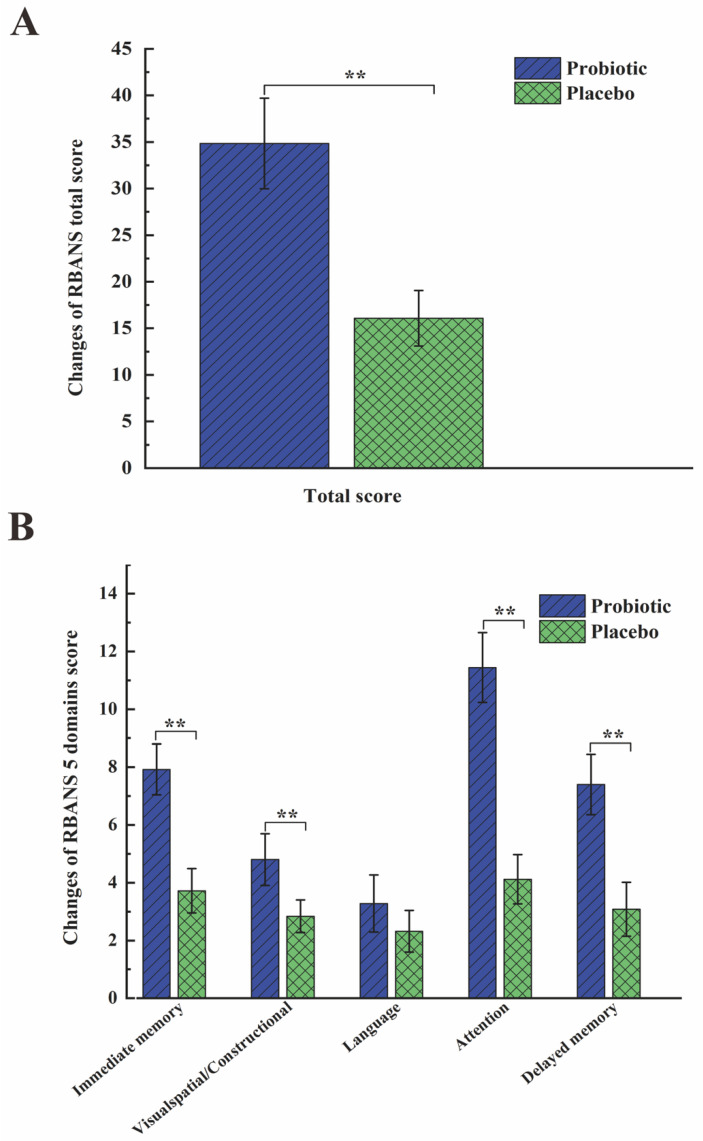
Changes in RBANS scores from baseline at 8 weeks. (**A**) Changes of RBANS total score from baseline at week 8. (**B**) Changes of RBANS total score from baseline at week 8. Values are indicated as mean and SE with error bars. ** *p* < 0.01.

Each subject underwent RBANS assessment at baseline and at week 8, and the effects of BB68S on cognitive functioning were evaluated (Table 2). BB68S intervention significantly enhanced the cognitive function total score by 18.89 points (95% CI 14.98 to 22.80, *p* < 0.0001). As shown in Table, the BB68S group had significantly higher scores in 4 domains than the control group after 8 weeks of intervention. The score differences (95% confidence intervals) between groups were 4.36 (2.95 to 5.76, *p* < 0.0001) for immediate memory, 2.01 (1.18 to 2.83, *p* < 0.0001) for visuospatial/constructional, 7.29 (4.77 to 9.80, *p* < 0.0001) for attention, and 4.28 (95% CI 2.26 to 6.30, *p* < 0.0001) for delayed memory. BB68S’s effect on language was nonsignificant (*p* = 0.141). Changes in RBANS scores after the intervention were calculated and are exhibited in Figure 3 for the two groups. Subjects in the BB68S group had significantly greater changes in total score and in almost all domains, except for the language domain.

### 3.3. Results of Gut Microbiota Composition

To determine the relationship between the improvement in cognitive function and the gut microbiota, we performed gut microbiome analysis for all participants. After the amplification and cloning of bacterial sequences, purified amplicons were sequenced for a total of 50 fecal samples. Finally, 1133 OTUs from all samples were annotated, which were divided into 13 phyla, 23 classes, 56 orders, 109 families, 283 genera, and 600 species.

#### 3.3.1. Results of Alpha-Diversity

After intervention, the BB68S group had increased Sob, Chao, Ace, and Shannon indexes and a reduced Simpson index, but not all changes were statistically significant (*p* > 0.05) (Table 3). This indicated that BB68S showed a tendency towards improving the alpha diversity of the gut microbiota but did not alter it significantly.

#### 3.3.2. Results of Beta-Diversity

**Figure 4:**
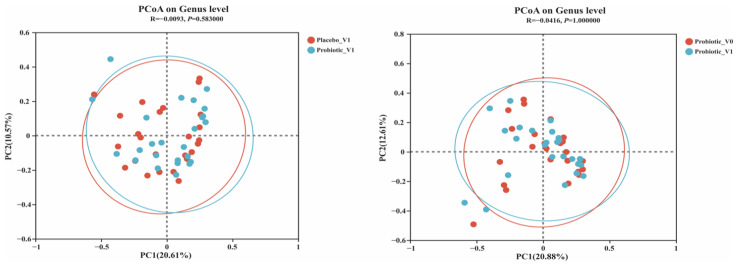
Principal coordinate analysis (PCoA) of the gut microbiota at the operational taxonomic unit (OTU) level. (**A**) Differences in V4 between the Placebo and Probiotic groups. (**B**) Differences between V0 and V1 in the Probiotic group. Placebo_V1: samples at V1 in the placebo group, Probiotic_V1: samples at V1 in the probiotic group, Probiotic_V0: samples at V0 in the probiotic group.

In Figure 4, the results of the principal coordinate analysis showed that the V0 and V1 regions of the probiotic group and the V1 regions between the BB68S and placebo groups did not differ significantly (*p* > 0.05). These results indicated that the BB68S intervention did not have a substantial impact on the beta diversity of the gut microbiota.

#### 3.3.3. Results of the Species Composition Analysis

**Figure 5:**
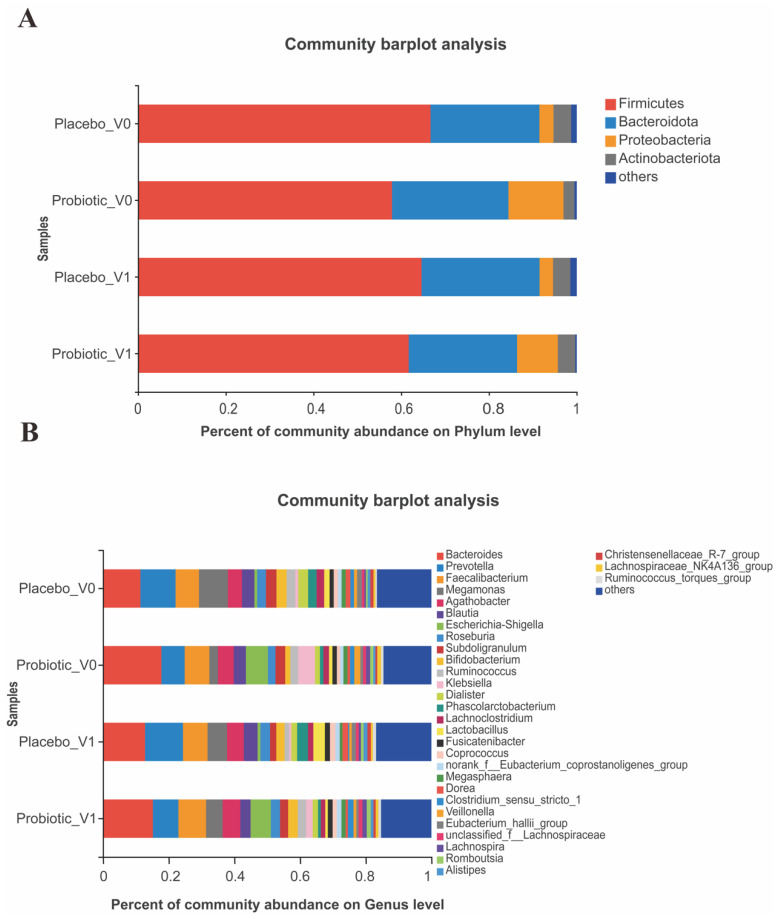

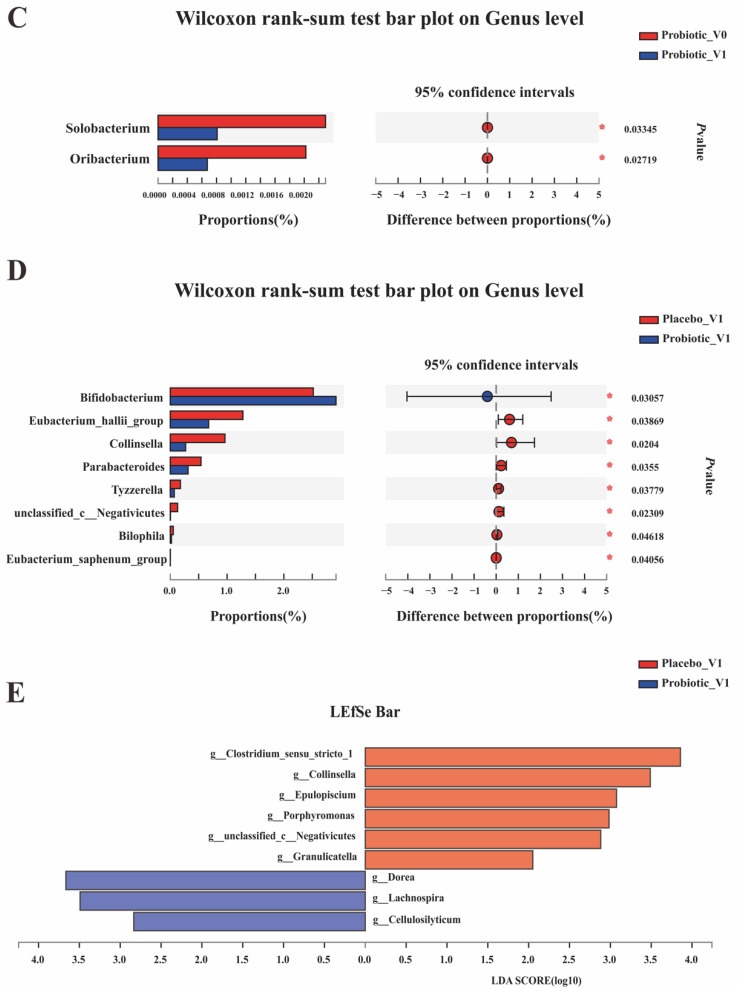
Effects of BB68S intervention on the composition of the gut microbiota. (**A**) Relative abundances of main phyla between groups. (**B**) Relative abundances of main genera ≥1% between groups. (**C**) Significantly different genera levels after intervention in the BB68S group. (**D**) Significantly different genera levels between 2 groups after intervention. (**E**) LEfSe analysis indicated significant differences between the 2 groups after intervention. * *p* ≤ 0.05. Placebo_V0: samples of the placebo group at baseline, Probiotic_V0: samples of the probiotic group at baseline, Placebo_V1: samples of the placebo group after intervention, Probiotic_V1: samples of the BB68S group after intervention.

Figure 5 displays the composition of the gut microbiota between the BB68S and placebo groups at the phylum and genus levels.

At the phylum level (Figure 5A), the gut microbiota mainly included Firmicutes, Bacteroidetes, Proteobacteria, and Actinobacteria. After intervention, the BB68S group had increased relative abundances of Firmicutes and Actinobacteria with a decreased relative abundance of Proteobacteria.

Figure 5B shows the bacteria with relative abundances of more than 1% at the genus level, including *Bacteroides*, *Prevotella*, *Faecalibacterium*, *Megamonas*, *Agathobacter*, *Blautia*, *Escherichia-Shigella*, *Roseburia*, *Subdoligranulum*, *Bifidobacterium*, *Ruminococcus*, *Klebsiella*, *Dialister*, *Phascolarctobacterium*, *Lachnoclostridium*, *Lactobacillus*, *Fusicatenibacter*, and *Coprococcus.*

Figure 5C,D exhibits the differences in the fecal bacterial population at the genus level. After BB68S intervention, the relative abundances of *Solobacterium* and *Oribacterium* decreased significantly (Figure 5C). Compared to the control, the BB68S group had a significantly higher relative abundance of *Bifidobacterium* and lower relative abundances of *Eubacterium_hallif_group*, *Collinsella*, *Parabacteroides*, *Tyzzerella*, *Bilophila*, *Eubacterium_saphenum_group*, and *unclassified_c_Negativicutes* after 8 weeks of intervention (Figure 5D).

In Figure 5E, we employed LEfSe analysis to further determine if different groups of bacterial taxa were specifically enriched after intervention. The results showed that the bacteria taxa significantly enriched in the control group were *Clostridium_sensu_stricto_1*, *Collinsella*, *Epulopiscium*, *Porphyromonas, unclassified_c_Negativicutes*, and *Granulicatella*, while in the BB68S group, these taxa were *Dorea*, *Lachnospira*, and *Cellulosilyticum*.

## 4. Discussion

This study was a randomized, double-blind, placebo-controlled trial conducted among healthy elderly people, showing the effects of probiotics on cognitive function in the healthy elderly. The subjects had good tolerance to the probiotic intervention in this study and no side effects were reported.

To our knowledge, among articles assessing the effect of probiotics on cognitive function, only two studies published to date have been conducted on the healthy elderly [35,36] and they were limited by the lack of consideration given to the subjects’ education level and lack of strict assessment criteria for cognitive health. In addition, the existing research tended to include some active and educated volunteers who cared about age-related memory problems and did not represent the general population. In a study of memory among middle-aged and elderly people, a relatively small but significant difference in the subjects’ education level between the two treatment groups impacted the results [37]. In our study, we recruited subjects with different education levels, which better represents the general population.

MoCA, developed by Nasreddine in 2004, is an assessment tool with 30 items used for rapid evaluation of cognitive function. Nine cognitive areas are assessed. The total score of the scale is 30 points. The higher the score, the better the global cognitive function. Its validity has been studied in various clinical settings [38,39,40,41,42]. In addition to being a concise cognitive assessment tool, MoCA is specific and useful to distinguish cognitive health from cognitive impairment. Based on MoCA, the criteria used to judge cognitive health are different for individuals with different education levels, which improves the screening sensitivity and effectiveness [43]. A total score of 19 points or more is considered representative of normal cognitive function among those who have been educated for less than or equal to 6 years, and scores of 22 and 24 points apply to those who have been educated for between 7 and 12 years and more than 12 years, respectively. In a study including Chinese healthy elderly with different levels of education, MoCA was sensitive, reliable, and highly accepted for the screening of cognitive function [43]. In our study, MoCA was used as a tool to cognitively screen healthy elderly people. Since we recruited the elderly without cognitive functional impairments in this study, rather than focusing on those who responded to the treatment effect, such as patients with neurological diseases, and strict screening tools (MoCA) with high sensitivity and effectiveness were used, our research is more applicable to a universal medical strategy for the elderly and has broader social significance.

In addition, this study used RBANS to evaluate the cognitive level of subjects. RBANS, a short assessment scale, was developed by Randolph in 1998 [44]. It contains 12 items, which are divided into 5 global areas that include immediate memory, visual space/structure, language, attention, and delayed memory. It is widely used in cognitive research at home and abroad to assess the cognitive level of normal people and patients, showing high reliability and validity [45,46,47,48]. In previous clinical studies, RBANS has been used to assess cognitive improvement after intervention and has good sensitivity [48]. The scale analysis for various aspects of brain functions shows the effect of the intervention on different cognitive domains.

In our study, BB68S intervention significantly improved cognitive function (total RBANS score increased by 18.89 points, *p* < 0.0001) compared to that of controls. Among the 12 items, list learning, story memory, figure copy, line orientation, coding, story recall, and figure recall scores of the probiotic group improved significantly. Among the 5 domains, only language did not significantly improve. All of the other four domains improved significantly in the BB68S group. The scores increased as follows: 4.36 points for immediate memory, 2.01 points for visuospatial/constructional, 7.19 points for attention, and 4.28 points for delayed memory (all *p*-values were less than 0.0001). In contrast, another study evaluating the effect of drinking lactic acid bacteria-fermented milk on memory only observed significant improvement in memory (the score increased by 4 points), with no change in total RBANS score and other domain scores [49]. *Bifidobacterium longum* BB68S significantly improved the cognitive function of healthy elderly people, providing strong evidence for future research and investigation.

Changes in the gut microbiota were essential to our study. Notably, the microbial composition of the probiotics group changed after the intervention, and the relative abundances of *Solobacterium* and *Oribacterium*, which cause inflammation, decreased significantly. Studies have shown that the relative abundances of *Solobacterium* and *Oribacterium* in the pro-inflammatory microbiota associated with colorectal cancer and reflux esophagitis were significantly reduced after the consumption of probiotics [50,51]. Based on Wilcoxon rank-sum and LEfSe analyses, many taxa showed differential abundance between the BB68S and control groups. The relative abundance of *Bifidobacterium* increased markedly after BB68S intervention. Some studies have shown that *Bifidobacterium* plays beneficial roles in the body, including regulating the gut microbiota and improving immune function [52]. The relative abundances of *Collinsella*, *Parabacteroides*, *Tyzzerella*, *Bilophila*, *Eubacterium_saphenum_group*, and *unclassified_c_Negativicutes* decreased significantly after BB68S intervention. *Collinsella* is an inflammation-related bacterium. Many studies have reported the association between the increased abundance of *Collinsella* and inflammatory diseases [53]. An increase in the abundance of *Parabacteroides* induced depression-like behavior in SAMP6 mice [54]. *Tyzzerella* has been negatively associated with 17-HAMD scores, playing a key role in metabolic disorders in patients with postpartum depression [55]. Hippocampal IL-1β levels have shown a positive correlation with the relative abundance of *Tyzzerella*, indicating that its increase might lead to neuroinflammation to a certain extent [56]. *Bilophila* is an obligate anaerobic pathobiont that is harmful to the hippocampus and cognitive behaviors [57]. In addition, *unclassified_c_Negativicutes* was found to be more abundant in patients suffering from Parkinson’s disease than in controls [58]. LEfSe analysis indicated that *Dorea* was enriched in the probiotic-treated group. These bacteria produce acetate and lactate that serve as substrates for butyrate production, and they are positively related to the immune response and negatively related to depression [59]. Many studies have shown that *Lachnospira* is negatively related to anxiety, depression, Parkinson’s disease, and psychiatric disorders [60,61,62]. The relative abundance of *Lachnospira* increased significantly following probiotic consumption according to the LEfSe analysis. *Porphyromonas gingivalis* is increasingly implicated in Alzheimer’s disease, cancer, and arthritis. The LEfSe results showed that probiotic intervention significantly reduced the relative abundance of the *Porphyromonas* genus [63].

The present study had some limitations that need to be addressed. First, our research lacked an analysis of the metabolites in the peripheral system and intestinal flora, and thus cannot explain the mechanism of action of the probiotics. In the coming research, we will attempt to carry out exploratory biomarker analysis to fully understand how probiotics improve the cognitive function of healthy elderly individuals. Second, although cognitive functions improved significantly, changes in some cognitive domains and intestinal flora were not significant. Eight weeks of study may not be sufficient to observe these results. Therefore, further research is needed with a longer duration. Third, although we instructed the subjects not to adjust their habitual diet during the intervention period, we did not strictly control their diet; therefore, we could not rule out the impact of dietary changes on gut microbiota. Studies with more rigorous designs (e.g., strict diet control) are needed in the future. Despite these limitations, our study also had some advantages. This study included subjects with diverse education levels and used highly sensitive screening tools specifically for different levels of education. We strictly and effectively evaluated cognitive function and measured the significant effects of probiotics on cognitive function with a short-term intervention among the healthy elderly.

In conclusion, our research showed that *Bifidobacterium longum* BB68S could improve cognitive function and has a beneficial regulatory effect on the gut microbiota in healthy elderly individuals. This study provides some evidence supporting probiotics as an alternative strategy to advance cognitive aging and promote healthy aging.

## Figures and Tables

**Table 1:** Demographic data of the study subjects.

	BB68S	Placebo	*p*-Value
Age (years)	64.10 ± 3.40	64.50 ± 3.79	0.67 ^1^
Sex			
Male (%)	12 (40)	13 (43)	0.77 ^2^
Female Male (%)	18 (60)	17 (57)	
Height (cm)	164.79 ± 8.18	165.09 ± 9.05	0.89 ^1^
Weight (kg)	67.13 ± 13.50	67.21 ± 12.50	0.98 ^1^
BMI (kg/m^2^)	24.55 ± 3.47	24.49 ± 3.03	0.95 ^1^
Education			
Elementary or less	10	10	
Junior-high school	13	12	
High school or more	7	8	
MoCA total score	23.03 ± 2.50	22.97 ± 2.43	0.92 ^1^
RBANS total score	186.90 ± 17.22	186.93 ± 15.62	0.99 ^1^

^1^ Independent sample’s *t*-test. ^2^ Chi-square test.

**Table 2:** The effects of BB68S on RBANS scores.

	Baseline		Week 8			
	Placebo (n = 25)	BB68S (n = 25)	Placebo (n = 25)	BB68S (n = 25)	Difference (95% CI)	*p*-Value
Total score	185.88 ± 16.77	186.88 ± 18.32	201.96 ± 17.14	221.72 ± 16.20	18.89 (14.98 to 22.80)	<0.0001
Immediate memory	31.56 ± 5.03	32.20 ± 5.69	35.28 ± 5.09	40.12 ± 4.35	4.36 (2.95 to 5.76)	<0.0001
List learning	20.28 ± 3.03	20.84 ± 3.82	22.56 ± 3.19	25.52 ± 4.00	2.49 (1.24 to 3.74)	<0.0001
Story memory	11.28 ± 3.54	11.36 ± 2.93	12.72 ± 3.49	14.60 ± 2.06	1.82 (0.81 to 2.84)	<0.0001
Visuospatial/Constructional	30.56 ± 4.08	30.68 ± 5.13	33.40 ± 3.33	35.48 ± 2.35	2.01 (1.18 to 2.83)	<0.0001
Figure copy	16.32 ± 2.69	16.48 ± 2.54	17.64 ± 2.18	19.04 ± 1.02	1.33 (0.62 to 2.04)	<0.0001
Line orientation	14.24 ± 2.85	14.20 ± 2.60	15.76 ± 2.33	16.44 ± 2.22	0.71 (0.03 to 1.39)	0.042
Language	29.76 ± 3.56	30.36 ± 4.39	32.08 ± 3.66	33.64 ± 3.87	1.09 (−0.38 to 2.56)	0.141
Picture naming	9.68 ± 0.75	9.60 ± 0.91	9.92 ± 0.28	9.76 ± 0.60	−0.14 (−0.36 to 0.09)	0.233
Semantic fluency	20.08 ± 3.37	20.76 ± 3.48	22.16 ± 3.65	23.88 ± 3.57	1.23 (−0.29 to 2.74)	0.11
Attention	55.44 ± 9.99	55.20 ± 9.80	59.56 ± 10.34	66.64 ± 8.68	7.29 (4.77 to 9.80)	<0.0001
Digit span	14.16 ± 1.95	14.12 ± 1.96	14.36 ± 1.85	14.44 ± 1.73	0.10 (−0.66 to 0.87)	0.785
Coding	41.28 ± 9.63	41.08 ± 10.21	45.20 ± 10.11	52.20 ± 8.40	7.17 (4.62 to 9.71)	<0.0001
Delayed memory	38.56 ± 4.91	38.44 ± 4.54	41.64 ± 5.10	45.84 ± 4.17	4.28 (2.26 to 6.30)	<0.0001
List recall	4.96 ± 2.23	4.00 ± 2.25	6.00 ± 2.18	6.60 ± 2.08	1.06 (−0.03 to 2.14)	0.056
List recognition	18.04 ± 2.17	18.28 ± 1.81	18.84 ± 1.80	18.72 ± 1.67	−0.25 (−1.03 to 0.54)	0.526
Story recall	6.84 ± 1.97	7.20 ± 2.10	7.44 ± 2.38	9.56 ± 2.18	1.96 (0.75 to 3.17)	0.002
Figure recall	8.72 ± 2.69	8.96 ± 2.49	9.36 ± 1.91	10.96 ± 2.61	1.53 (0.29 to 2.77)	0.017

Mean ± SD is used to indicate values. Effects of BB68S are indicated by the difference (95% CI) between the two groups, calculated from the generalized linear model (GLM).

**Table 3:** The effects of BB68S on the α-diversity.

	Baseline		Week 8		^a^ *p*-Value	^b^ *p*-Value
	Placebo	BB68S	Placebo	BB68S		
Sobs	229.44 ± 83.66	233.96 ± 90.34	236.32 ± 73.97	241.32 ± 91.81	0.76	0.67
Ace	282.86 ± 98.31	276.57 ± 106.21	278.72 ± 87.53	286.95 ± 106.95	0.67	0.63
Chao	284.75 ± 97.13	273.67 ± 112.03	280.71 ± 84.41	293.49 ± 114.6	0.56	0.56
Shannon	3.28 ± 0.65	3.22 ± 0.97	3.29 ± 0.59	3.22 ± 0.77	0.95	0.91
Simpson	0.1 ± 0.07	0.14 ± 0.22	0.11 ± 0.08	0.11 ± 0.09	0.77	0.95

Mean ± SD is used to indicate values; ^a^ *p*-values indicate the change from baseline to Week 8 for the BB68S group; ^b^ *p*-values indicate the difference between the 2 groups at Week 8; ^a^ and ^b^ were both calculated using Wilcoxon rank-sum tests.

## Data Availability

Data are contained within the article.
